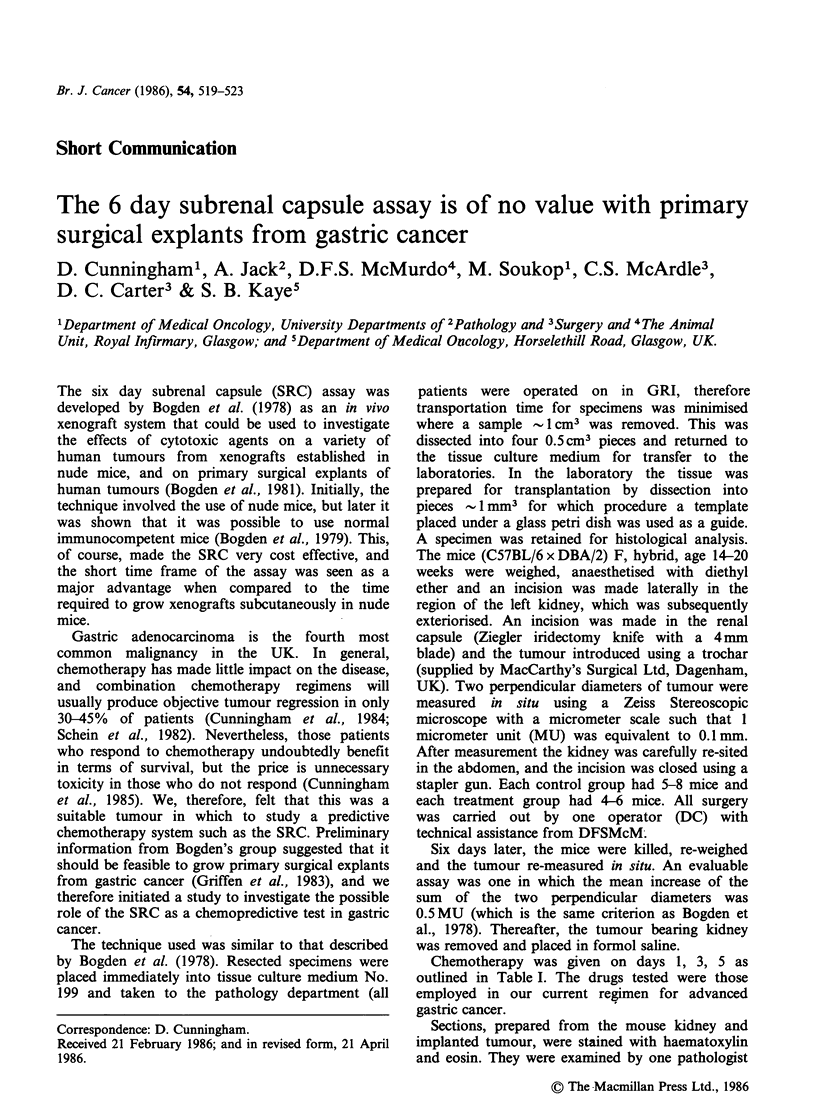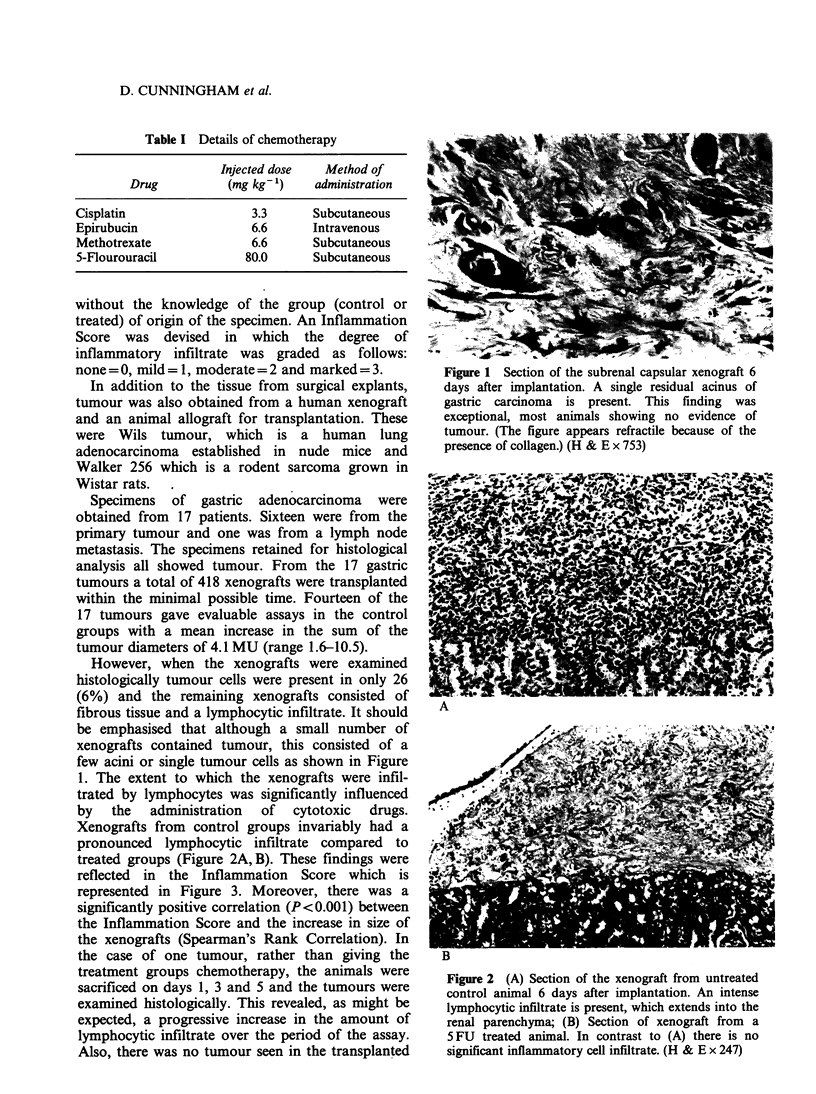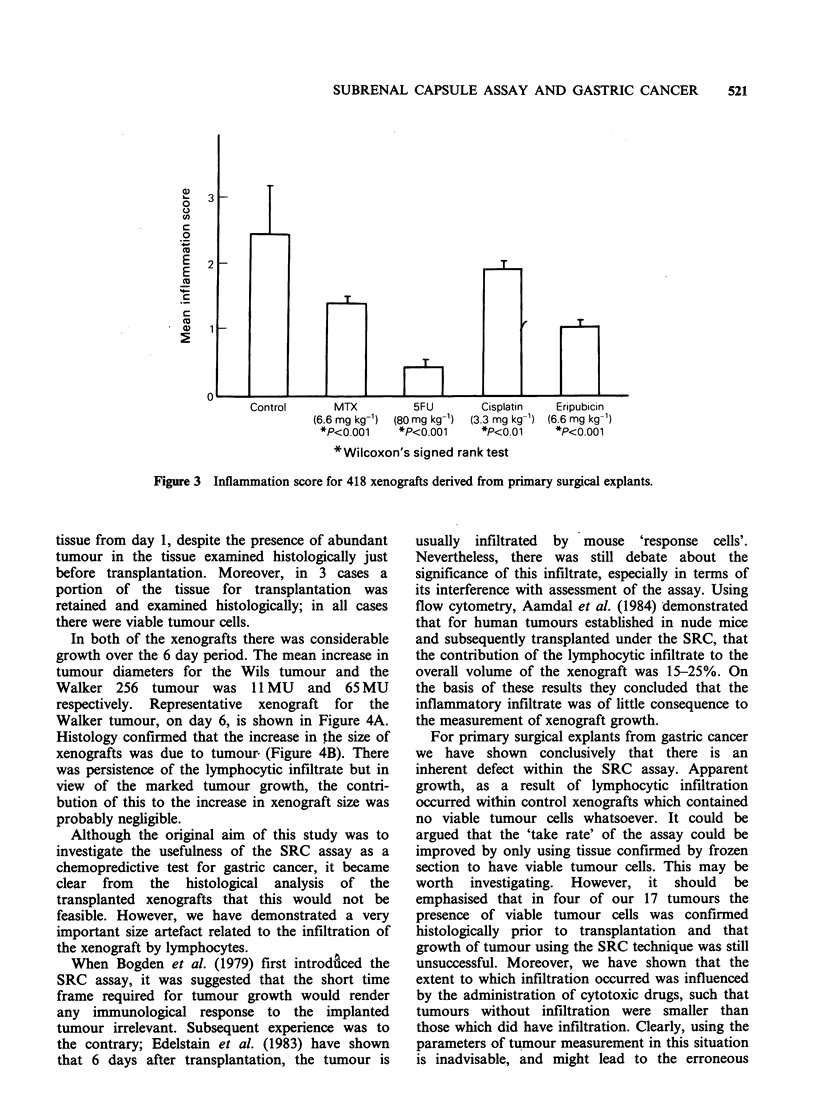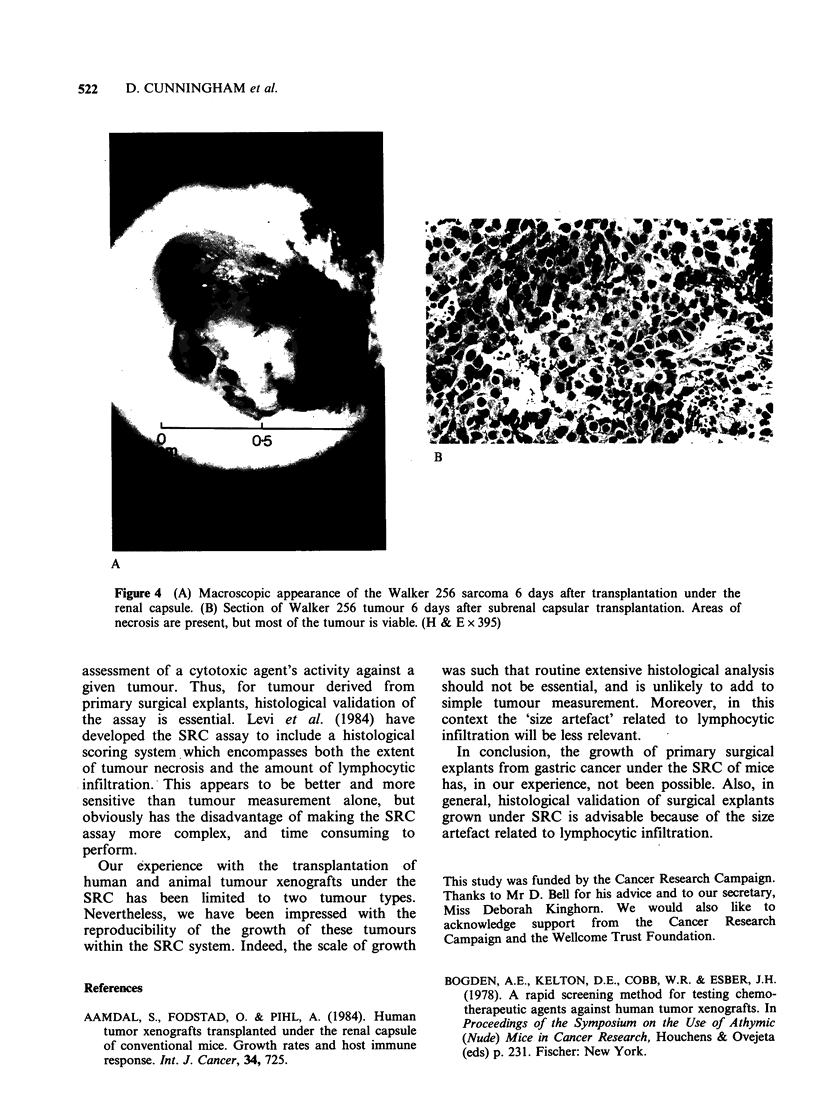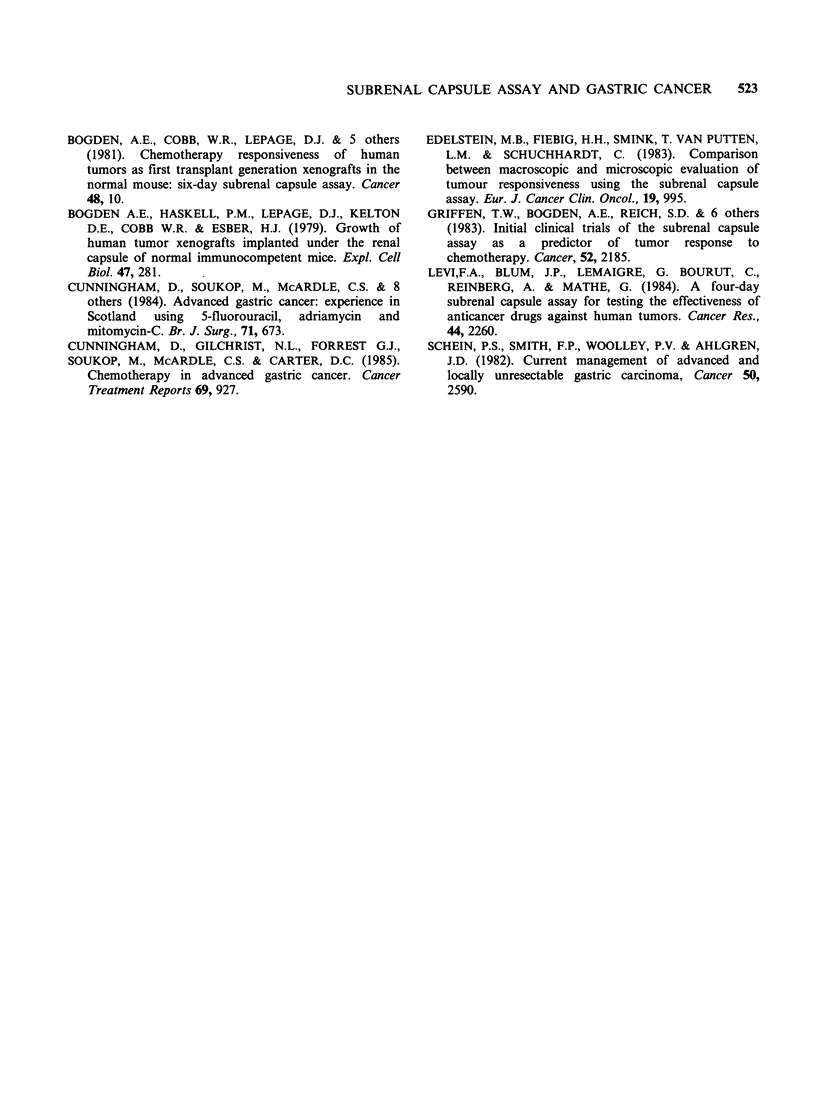# The 6 day subrenal capsule assay is of no value with primary surgical explants from gastric cancer.

**DOI:** 10.1038/bjc.1986.207

**Published:** 1986-09

**Authors:** D. Cunningham, A. Jack, D. F. McMurdo, M. Soukop, C. S. McArdle, D. C. Carter, S. B. Kaye

## Abstract

**Images:**


Br. J. Cancer (1986), 54, 519–523

### Short Communication

### The 6 day subrenal capsule assay is of no value with primary surgical explants from gastric cancer

D. Cunningham[1], A. Jack[2], D.F.S. McMurdo[4], M. Soukop[1], C.S. McArdle[3], D. C. Carter[3] & S. B. Kaye[5]

[1]*Department of Medical Oncology, University Departments of* [2]*Pathology and* [3]*Surgery and* [4]*The Animal Unit, Royal Infirmary, Glasgow; and* [5]*Department of Medical Oncology, Horselethill Road, Glasgow, UK.*

The six day subrenal capsule (SRC) assay was developed by Bogden *et al.* (1978) as an *in vivo* xenograft system that could be used to investigate the effects of cytotoxic agents on a variety of human tumours from xenografts established in nude mice, and on primary surgical explants of human tumours (Bogden *et al.*, 1981). Initially, the technique involved the use of nude mice, but later it was shown that it was possible to use normal immunocompetent mice (Bogden *et al.*, 1979). This, of course, made the SRC very cost effective, and the short time frame of the assay was seen as a major advantage when compared to the time required to grow xenografts subcutaneously in nude mice.

Gastric adenocarcinoma is the fourth most common malignancy in the UK. In general, chemotherapy has made little impact on the disease, and combination chemotherapy regimens will usually produce objective tumour regression in only 30–45% of patients (Cunningham *et al.*, 1984; Schein *et al.*, 1982). Nevertheless, those patients who respond to chemotherapy undoubtedly benefit in terms of survival, but the price is unnecessary toxicity in those who do not respond (Cunningham *et al.*, 1985). We, therefore, felt that this was a suitable tumour in which to study a predictive chemotherapy system such as the SRC. Preliminary information from Bogden's group suggested that it should be feasible to grow primary surgical explants from gastric cancer (Griffen *et al.*, 1983), and we therefore initiated a study to investigate the possible role of the SRC as a chemopredictive test in gastric cancer.

The technique used was similar to that described by Bogden *et al.* (1978). Resected specimens were placed immediately into tissue culture medium No. 199 and taken to the pathology department (all patients were operated on in GRI, therefore transportation time for specimens was minimised where a sample $\sim 1\,cm^3$ was removed. This was dissected into four $0.5\,cm^3$ pieces and returned to the tissue culture medium for transfer to the laboratories. In the laboratory the tissue was prepared for transplantation by dissection into pieces $\sim 1\,mm^3$ for which procedure a template placed under a glass petri dish was used as a guide. A specimen was retained for histological analysis. The mice (C57BL/6 × DBA/2) F, hybrid, age 14–20 weeks were weighed, anaesthetised with diethyl ether and an incision was made laterally in the region of the left kidney, which was subsequently exteriorised. An incision was made in the renal capsule (Ziegler iridectomy knife with a 4 mm blade) and the tumour introduced using a trochar (supplied by MacCarthy's Surgical Ltd, Dagenham, UK). Two perpendicular diameters of tumour were measured *in situ* using a Zeiss Stereoscopic microscope with a micrometer scale such that 1 micrometer unit (MU) was equivalent to 0.1 mm. After measurement the kidney was carefully re-sited in the abdomen, and the incision was closed using a stapler gun. Each control group had 5–8 mice and each treatment group had 4–6 mice. All surgery was carried out by one operator (DC) with technical assistance from DFSMcM.

Six days later, the mice were killed, re-weighed and the tumour re-measured *in situ*. An evaluable assay was one in which the mean increase of the sum of the two perpendicular diameters was 0.5 MU (which is the same criterion as Bogden et al., 1978). Thereafter, the tumour bearing kidney was removed and placed in formol saline.

Chemotherapy was given on days 1, 3, 5 as outlined in Table I. The drugs tested were those employed in our current regimen for advanced gastric cancer.

Sections, prepared from the mouse kidney and implanted tumour, were stained with haematoxylin and eosin. They were examined by one pathologist

Correspondence: D. Cunningham.
Received 21 February 1986; and in revised form, 21 April 1986.

© The Macmillan Press Ltd., 1986

**Table I** Details of chemotherapy

| *Drug* | *Injected dose* $(mg\ kg^{-1})$ | *Method of administration* |
|---|---|---|
| Cisplatin | 3.3 | Subcutaneous |
| Epirubucin | 6.6 | Intravenous |
| Methotrexate | 6.6 | Subcutaneous |
| 5-Flourouracil | 80.0 | Subcutaneous |

without the knowledge of the group (control or treated) of origin of the specimen. An Inflammation Score was devised in which the degree of inflammatory infiltrate was graded as follows: none = 0, mild = 1, moderate = 2 and marked = 3.

In addition to the tissue from surgical explants, tumour was also obtained from a human xenograft and an animal allograft for transplantation. These were Wils tumour, which is a human lung adenocarcinoma established in nude mice and Walker 256 which is a rodent sarcoma grown in Wistar rats.

Specimens of gastric adenocarcinoma were obtained from 17 patients. Sixteen were from the primary tumour and one was from a lymph node metastasis. The specimens retained for histological analysis all showed tumour. From the 17 gastric tumours a total of 418 xenografts were transplanted within the minimal possible time. Fourteen of the 17 tumours gave evaluable assays in the control groups with a mean increase in the sum of the tumour diameters of 4.1 MU (range 1.6–10.5).

However, when the xenografts were examined histologically tumour cells were present in only 26 (6%) and the remaining xenografts consisted of fibrous tissue and a lymphocytic infiltrate. It should be emphasised that although a small number of xenografts contained tumour, this consisted of a few acini or single tumour cells as shown in Figure 1. The extent to which the xenografts were infiltrated by lymphocytes was significantly influenced by the administration of cytotoxic drugs. Xenografts from control groups invariably had a pronounced lymphocytic infiltrate compared to treated groups (Figure 2A, B). These findings were reflected in the Inflammation Score which is represented in Figure 3. Moreover, there was a significantly positive correlation ($P<0.001$) between the Inflammation Score and the increase in size of the xenografts (Spearman's Rank Correlation). In the case of one tumour, rather than giving the treatment groups chemotherapy, the animals were sacrificed on days 1, 3 and 5 and the tumours were examined histologically. This revealed, as might be expected, a progressive increase in the amount of lymphocytic infiltrate over the period of the assay. Also, there was no tumour seen in the transplanted

**Figure 1** Section of the subrenal capsular xenograft 6 days after implantation. A single residual acinus of gastric carcinoma is present. This finding was exceptional, most animals showing no evidence of tumour. (The figure appears refractile because of the presence of collagen.) (H & E × 753)

A

B

**Figure 2** (A) Section of the xenograft from untreated control animal 6 days after implantation. An intense lymphocytic infiltrate is present, which extends into the renal parenchyma; (B) Section of xenograft from a 5 FU treated animal. In contrast to (A) there is no significant inflammatory cell infiltrate. (H & E × 247)

**Figure 3** Inflammation score for 418 xenografts derived from primary surgical explants.

tissue from day 1, despite the presence of abundant tumour in the tissue examined histologically just before transplantation. Moreover, in 3 cases a portion of the tissue for transplantation was retained and examined histologically; in all cases there were viable tumour cells.

In both of the xenografts there was considerable growth over the 6 day period. The mean increase in tumour diameters for the Wils tumour and the Walker 256 tumour was 11 MU and 65 MU respectively. Representative xenograft for the Walker tumour, on day 6, is shown in Figure 4A. Histology confirmed that the increase in the size of xenografts was due to tumour (Figure 4B). There was persistence of the lymphocytic infiltrate but in view of the marked tumour growth, the contribution of this to the increase in xenograft size was probably negligible.

Although the original aim of this study was to investigate the usefulness of the SRC assay as a chemopredictive test for gastric cancer, it became clear from the histological analysis of the transplanted xenografts that this would not be feasible. However, we have demonstrated a very important size artefact related to the infiltration of the xenograft by lymphocytes.

When Bogden *et al.* (1979) first introduced the SRC assay, it was suggested that the short time frame required for tumour growth would render any immunological response to the implanted tumour irrelevant. Subsequent experience was to the contrary; Edelstain *et al.* (1983) have shown that 6 days after transplantation, the tumour is usually infiltrated by mouse 'response cells'. Nevertheless, there was still debate about the significance of this infiltrate, especially in terms of its interference with assessment of the assay. Using flow cytometry, Aamdal *et al.* (1984) demonstrated that for human tumours established in nude mice and subsequently transplanted under the SRC, that the contribution of the lymphocytic infiltrate to the overall volume of the xenograft was 15–25%. On the basis of these results they concluded that the inflammatory infiltrate was of little consequence to the measurement of xenograft growth.

For primary surgical explants from gastric cancer we have shown conclusively that there is an inherent defect within the SRC assay. Apparent growth, as a result of lymphocytic infiltration occurred within control xenografts which contained no viable tumour cells whatsoever. It could be argued that the 'take rate' of the assay could be improved by only using tissue confirmed by frozen section to have viable tumour cells. This may be worth investigating. However, it should be emphasised that in four of our 17 tumours the presence of viable tumour cells was confirmed histologically prior to transplantation and that growth of tumour using the SRC technique was still unsuccessful. Moreover, we have shown that the extent to which infiltration occurred was influenced by the administration of cytotoxic drugs, such that tumours without infiltration were smaller than those which did have infiltration. Clearly, using the parameters of tumour measurement in this situation is inadvisable, and might lead to the erroneous

**Figure 4** (A) Macroscopic appearance of the Walker 256 sarcoma 6 days after transplantation under the renal capsule. (B) Section of Walker 256 tumour 6 days after subrenal capsular transplantation. Areas of necrosis are present, but most of the tumour is viable. (H & E × 395)

assessment of a cytotoxic agent's activity against a given tumour. Thus, for tumour derived from primary surgical explants, histological validation of the assay is essential. Levi *et al.* (1984) have developed the SRC assay to include a histological scoring system which encompasses both the extent of tumour necrosis and the amount of lymphocytic infiltration. This appears to be better and more sensitive than tumour measurement alone, but obviously has the disadvantage of making the SRC assay more complex, and time consuming to perform.

Our experience with the transplantation of human and animal tumour xenografts under the SRC has been limited to two tumour types. Nevertheless, we have been impressed with the reproducibility of the growth of these tumours within the SRC system. Indeed, the scale of growth was such that routine extensive histological analysis should not be essential, and is unlikely to add to simple tumour measurement. Moreover, in this context the 'size artefact' related to lymphocytic infiltration will be less relevant.

In conclusion, the growth of primary surgical explants from gastric cancer under the SRC of mice has, in our experience, not been possible. Also, in general, histological validation of surgical explants grown under SRC is advisable because of the size artefact related to lymphocytic infiltration.

This study was funded by the Cancer Research Campaign. Thanks to Mr D. Bell for his advice and to our secretary, Miss Deborah Kinghorn. We would also like to acknowledge support from the Cancer Research Campaign and the Wellcome Trust Foundation.

**References**

AAMDAL, S., FODSTAD, O. & PIHL, A. (1984). Human tumor xenografts transplanted under the renal capsule of conventional mice. Growth rates and host immune response. *Int. J. Cancer*, **34,** 725.

BOGDEN, A.E., KELTON, D.E., COBB, W.R. & ESBER, J.H. (1978). A rapid screening method for testing chemotherapeutic agents against human tumor xenografts. In *Proceedings of the Symposium on the Use of Athymic (Nude) Mice in Cancer Research,* Houchens & Ovejeta (eds) p. 231. Fischer: New York.

BOGDEN, A.E., COBB, W.R., LEPAGE, D.J. & 5 others (1981). Chemotherapy responsiveness of human tumors as first transplant generation xenografts in the normal mouse: six-day subrenal capsule assay. *Cancer* **48,** 10.

BOGDEN A.E., HASKELL, P.M., LEPAGE, D.J., KELTON D.E., COBB W.R. & ESBER, H.J. (1979). Growth of human tumor xenografts implanted under the renal capsule of normal immunocompetent mice. *Expl. Cell Biol.* **47,** 281.

CUNNINGHAM, D., SOUKOP, M., McARDLE, C.S. & 8 others (1984). Advanced gastric cancer: experience in Scotland using 5-fluorouracil, adriamycin and mitomycin-C. *Br. J. Surg.,* **71,** 673.

CUNNINGHAM, D., GILCHRIST, N.L., FORREST G.J., SOUKOP, M., McARDLE, C.S. & CARTER, D.C. (1985). Chemotherapy in advanced gastric cancer. *Cancer Treatment Reports* **69,** 927.

EDELSTEIN, M.B., FIEBIG, H.H., SMINK, T. VAN PUTTEN, L.M. & SCHUCHHARDT, C. (1983). Comparison between macroscopic and microscopic evaluation of tumour responsiveness using the subrenal capsule assay. *Eur. J. Cancer Clin. Oncol.,* **19,** 995.

GRIFFEN, T.W., BOGDEN, A.E., REICH, S.D. & 6 others (1983). Initial clinical trials of the subrenal capsule assay as a predictor of tumor response to chemotherapy. *Cancer,* **52,** 2185.

LEVI,F.A., BLUM, J.P., LEMAIGRE, G. BOURUT, C., REINBERG, A. & MATHE, G. (1984). A four-day subrenal capsule assay for testing the effectiveness of anticancer drugs against human tumors. *Cancer Res.,* **44,** 2260.

SCHEIN, P.S., SMITH, F.P., WOOLLEY, P.V. & AHLGREN, J.D. (1982). Current management of advanced and locally unresectable gastric carcinoma, *Cancer* **50,** 2590.